# Warmer temperatures increase egg laying and egg hatching frequency in the invasive freshwater snail *Physa gyrina* from Pennsylvania, USA

**DOI:** 10.1007/s11356-025-37170-0

**Published:** 2025-11-14

**Authors:** Peter P. Fong, Morgan P. Nieman

**Affiliations:** Department of Biology, Gettysburg College, Gettysburg, PA 17325 USA

**Keywords:** Climate change, Wastewater, Freshwater snails, Antidepressant, Aquatic

## Abstract

Climate change continues to impact populations of organisms and to affect physiological adaptations to their environments. Freshwater snails have been so impacted globally, not only by climate change, but by the concomitant exposure to environmental pollutants like human antidepressants released from wastewater treatment plants. To test the effect of climate change and antidepressants, we exposed the freshwater snail, *Physa gyrina* to three temperatures (12 °C, 20 °C, and 25 °C) and to a concentration of the antidepressant fluoxetine, known to modulate snail behavior, and measured time to egg laying and egg hatching. Snails exposed to 20 °C and 25 °C laid significantly more egg masses sooner than snails at 12 °C. Embryos hatched from egg masses significantly sooner at the two warmer temperatures than those at 12 °C. Exposure to fluoxetine had only a minor effect on the timing of egg laying and no effect on the timing of egg hatching. Our findings suggest that warmer temperatures may be more important in modulating reproduction in *P. gyrina* than fluoxdetine fluoxetine. Since this species is invasive in Europe, we discuss our results in terms of the possible consequences of climate change on the potential geographical spread of invasive species.

## Introduction

Climate change has had profound effects on the distribution and abundance of different populations of organisms in different environments around the world (Fonturbel et al. 2021; McCarty 2002). In aquatic environments, warming water temperatures have been shown to affect physical and biological variables such as levels of dissolved oxygen, pH, algal blooms (Woodward et al. 2010; Woolway et al. 2022) as well as the expansion of species’ ranges into new habitats (Byers et al. 2013; Pinsky et al. 2020). Climate change has had an impact on biological invasions (Rahel and Olden 2008; Crooks et al. 2011; Teles et al. 2022), in many cases facilitating their establishment or allowing for expansion of already established invasive species. Increasing water temperature has also affected the behavior and physiology of aquatic invasive species and their interactions with native species, and how constraints imposed by environmental conditions affect each (Rahel and Olden 2008; Havel et al. 2015).

Climate change has potential to exacerbate/influence environmental pollution, and when combined with other pollutants like human antidepressants released from wastewater treatment plants, these multiple stressors could possibly affect a number of behaviors in aquatic snails including locomotion (Ford et al. 2018; Fong et al. 2019; Ziegler et al. 2021), habitat choice/boldness (Hedgespeth et al. 2018) and reproduction (Gust et al. 2009; Henry et al. 2022).

The freshwater snail *Physa gyrina* (Say, 1821) is common in lakes and streams in its native range in North America (Jokinen 1992), but it is invasive in Great Britain (Anderson 1996). Like many species in the genus *Physa*, *P. gyrina* can live on a variety of substrates including mud, sand, rock, and vegetation. It is a generalist feeder consuming algae, vascular plants, and organic material (Dillon 2024). In the present study, we measured two reproduction end points (egg laying and egg hatching) in snails collected from a spring-fed stream in south central Pennsylvania, USA where the temperature is a constant 12°C. We exposed snails to three temperatures (12°, 20°, and 25 °C) and the antidepressant fluoxetine (hereafter FLX) at a concentration (3.45 µg/L), known to affect reproduction in freshwater gastropods (Gust et al. 2009).

## Materials and methods

Snails (*Physa gyrina,* 7–12 mm shell length) were collected from a spring-fed creek in Boiling Springs, PA, USA (40.1498° N, 77.1283° W) and transported to the lab at Gettysburg College. The temperature of this stream is a constant 12 °C throughout the year, and populations of snails and other benthic macroinvertebrates have been monitored by one of us (PPF) since 1994. At this site, snails are continuously reproductive with egg masses laid every month of the year. Experiments began on the same days of collection (no acclimation). Snails were placed in plastic dishes (4.5” × 3.0”, Joshsfrogs.com; one snail per dish) containing 200 ml of spring water (IGA Natural Spring Water, pH 7.73, dissolved oxygen: 8.09 mg/L, conductivity 90.2 µS/cm**)** at three temperatures (12°, 20°, and 25 °C, n=15 per group).

The 12 °C temperature was selected because it is year-round temperature in Boiling Springs. The intermediate temperature (20 °C) corresponds to a moderate springtime temperature in our local, non-spring creeks, and 25 °C would be the maximum summertime temperature in our local creeks that are also not spring fed. Snails were not fed during all experiments, and we monitored them daily for egg mass production, for hatching time of embryos from each egg mass, and counted the number of embryos in each egg mass. If a snail laid an egg mass, we transferred the snail to a new dish containing 200 ml of spring water so that any new egg masses would be separated from previous egg masses. Water was changed in all dishes every 5 days until the end of the snail egg laying experiment (duration 24 days), but we continued to monitor all egg masses until hatching. In a second experiment, snails were also exposed to a nominal concentration of fluoxetine hydrochloride (FLX, 3.45 µg/L; Sigma Chemical Co., CAS Number: 56296–78-7) at each of the three temperatures (n = 12 per group, one snail per dish). We selected this concentration due to the effects of FLX from previous published reports on other freshwater snails. Gust et al. (2009) reported that FLX (3.7 µg/L) increased the number of embryos of the New Zealand mud snail *Potamopyrgus antipodarum* also at comparable temperatures.

### Statistical analysis

For time data (e.g., days to egg laying), normality was tested using the D’Agostino-Pearson test and variance homogeneity was confirmed by Bartlett’s test. Non-normal data were transformed with Box-Cox transformation (Weesa 2021). Mean differences between temperature groups were tested by one-way ANOVA with Tukey’s post hoc test. Differences in egg laying and embryo hatching frequency were tested with Fisher’s Exact Test and 3 × 2 contingency tables with Cramer’s V test of association for combined effects of temperature and FLX exposure.

## Results

### Mortality

Overall mortality was low (8.54%, including 0% and 5.1% in the controls for the first and second experiments respectively).

#### Experiment #1: The effect of three temperatures on egg laying and hatching

**Fig. 1:**
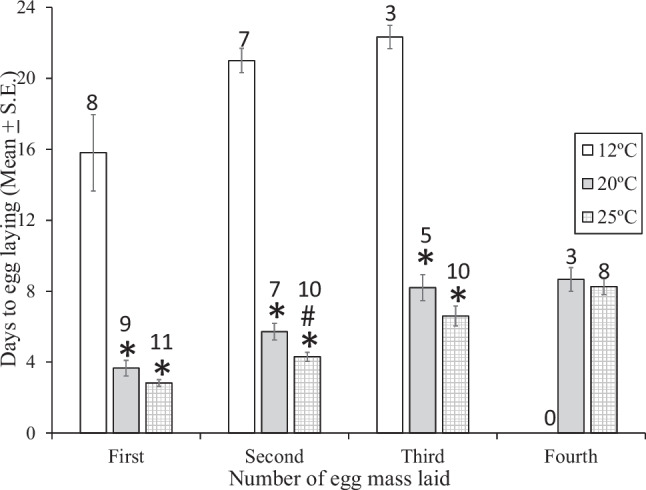
Mean (± S.E.) time in days for *Physa gyrina* snails to lay each of four egg masses at three temperatures (12°C, 20 °C, and 25° C). *: *p* < 0.05 vs. 12 °C, #: *p* < 0.05 vs. 20 °C, Tukey’s post hoc test following one-way ANOVA of Box-Cox transformed data. Number of snails laying eggs indicated above each bar

Snails laid from one to four egg masses over the course of the first experiment, but snails in 12 °C laid a maximum of only three egg masses over the course of 23 days whereas snails in both 20 °C and 25 °C degrees laid up to four egg masses in as short as 9–10 days. Snails in 20 °C and 25 °C began laying egg masses within three days of culture, but it took snails in 12 °C water 18 days to begin egg laying. The mean time until the first egg laying was significantly shorter for those in 20 °C and 25 °C compared with those in 12 °C (one-way ANOVA, *p* < 0.0001; Tukey’s *p* < 0.01 for both comparisons; Fig. 1, Table 1). Some snails in 20 °C and 25 °C continued to lay up to four egg masses over the duration of the first experiment (Fig. 1). Similarly, snails in in both 20 °C and 25 °C took a shorter time to lay a second egg mass than those in 12^o^C (one-way ANOVA, *p* < 0.0001), Tukey’s *p* < 0.01 for all three comparisons 12° vs. 20° and 12° vs. 25° and 20° vs. 25°; Fig. 1}. Snails in 20 °C and 25 °C also took significantly less time to lay a third egg mass compared to snails in 12 °C (one-way ANOVA, *p* < 0.0001, Tukey’s *p* < 0.01 for both comparisons 12° vs. 20° and 12° vs. 25°; Fig. 1, Table 1). We did not analyze the data for the fourth egg mass since snails in 12 °C did not produce any.

**Table 1:** ANOVA table for mean time (days) to egg laying for the first, second, and third egg masses by *Physa gyrina* when exposed to three temperatures (12 °C, 20 °C, and 25 °C). Data were Box-Cox transformed

first egg mass					
Source	SS	DF	MS	F	*p*-value
Temperature	1.54	2	0.77	19.93	**<0.0001**
Error	0.968	25	0.038		
Total	2.51	27			
Tukey's post-hoc test Comparison	*p*-value				
12^o^C vs. 20^o^C	<0.01				
12^o^C vs. 20^o^C	<0.01				
second egg mass					
Temperature	0.999	2	0.499	106.62	**<0.0001**
Error	0.098	21	0.0004		
Total	1.09	23			
Tukey's post-hoc test Comparison	*p*-value				
12^o^C vs. 20^o^C	<0.01				
12^o^C vs. 20^o^C	<0.01				
20^o^C vs. 25^o^C	<0.01				
third egg mass					
Tempeture	0.252	2	0.126	21.7	**<0001**
Error	0.08	15	0.005		
Total	0.34	17			
Tukey’s post-hoc testComparison	*p*-value				
12^o^C vs. 20^o^C	<0.01				
12^o^C vs. 20^o^C	<0.01				

Warmer temperatures resulted in a higher frequency of egg laying. Snails in 25 °C laid more egg masses and on multiple occasions than those in 12 °C. Egg laying frequency was higher in snails at 20° than at 12 °C, and higher in snails at 25 °C than at 20 °C.

**Fig. 2:**
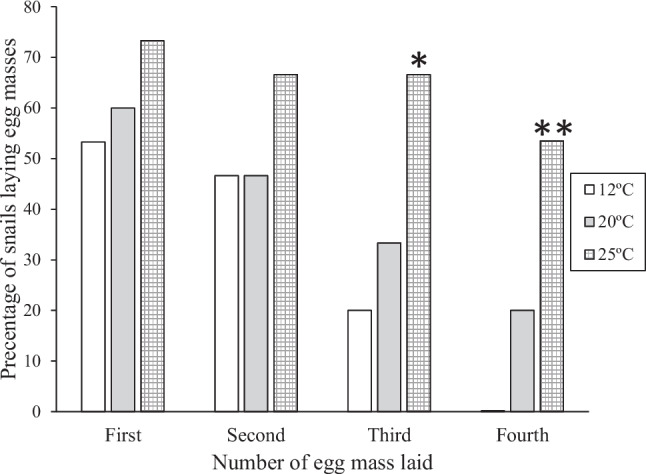
Percentage of snails laying each of four egg masses at three temperatures (12°C, 20 °C, and 25 °C). *: *p* < 0.01, **: *p* < 0.002 Fisher’s Exact Test vs. 12°C. Number of snails laying egg masses are the same as indicated in Fig. 1

The difference in egg laying frequency was significant for egg masses three and four (Fisher’s Exact Test, *p* < 0.01–0.002; Fig. 2).

**Fig. 3:**
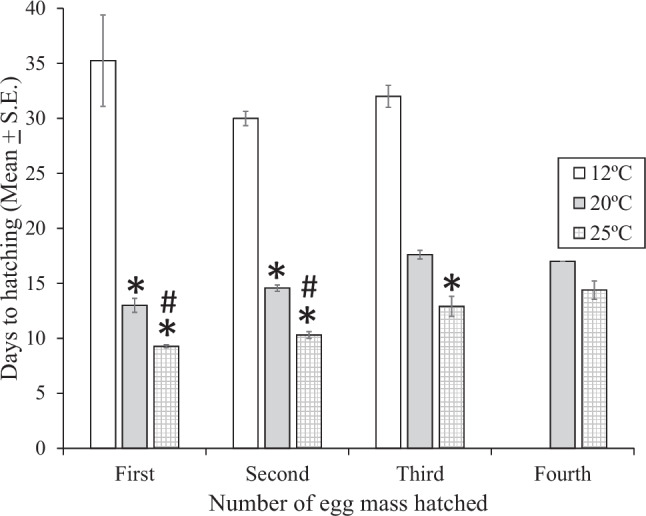
Mean (± S.E.). time in days for embryos of *Physa gyrina* to hatch from each of four egg masses at three temperatures (12°C, 20 °C, and 25° C). *: *p* < 0.01 vs. 12 °C, #: *p* < 0.01 vs. 20 °C, Tukey’s post hoc test following one-way ANOVA on Box-Cox transformed data. Number of egg masses are the same as the number of snails indicated in Fig. 1

Similar to the timing of egg laying, the timing for embryos within egg masses to hatch was influenced by temperature. At 20 °C or 25 °C, egg masses took a significantly shorter time to hatch, than at 12 °C (Fig. 3). This was the case for snails laying from one to three egg masses. The ANOVA results are shown in Table 2. For hatching from the first egg mass (one-way ANOVA, F(2, 25) = 273.45, *p* < 0.0001, Tukey’s *p* < 0.01) for all three comparisons (12 °C vs. 20 °C, 12 °C vs. 25 °C, and 20 °C vs. 25 °C). For hatching from the second (one-way F(2, 21) = 198.65, *p* < 0.0001, Tukey’s *p* < 0.01) for all three comparisons (12 °C vs. 20 °C, 12 °C vs. 25 °C, and 20 °C vs. 25 °C). For hatching from the third egg mass, only those in 25 °C hatched significantly faster than those in 12 °C (one-way F(2, 15) = 19.34, *p* < 0.0001, Tukey’s *p* < 0.01).

**Table 2:** ANOVA table for mean time (days) to egg hatching of the first, second, and third egg masses by embryos of *Physa gyrina* when exposed to three temperatures (12 °C, 20 °C, and 25 °C). Data were Box-Cox transformed

first egg mass					
Source	SS	DF	MS	F	*p*-value
Temperature	0.004	2	0.002	273.45	**<0.0001**
Error	0.0002	25	0.000008		
Total	0.0048	27			
Tukey's post-hoc test Comparison	*p*-value				
12^o^C vs. 20^o^C	<0.01				
12^o^C vs. 20^o^C	<0.01				
20^o^C vs. 25^o^C	<0.01				
second egg mass					
Temperature	0.0028	2	0.0014	198.65	**<0.0001**
Error	0.00014	21	0.000007		
Total	0.003	23			
Tukey's post-hoc test Comparison	*p*-value				
12^o^C vs. 20^o^C	<0.01				
12^o^C vs. 20^o^C	<0.01				
20^o^C vs. 25^o^C	<0.01				
third egg mass					
Temperature	0.0009	2	0.0004	19.34	**<0.0001**
Error	0.0003	15	0.00002		
Total	0.0013	17			
Tukey's post-hoc test Comparison	*p*-value				
12^o^C vs. 25^o^C	<0.01				

**Fig. 4:**
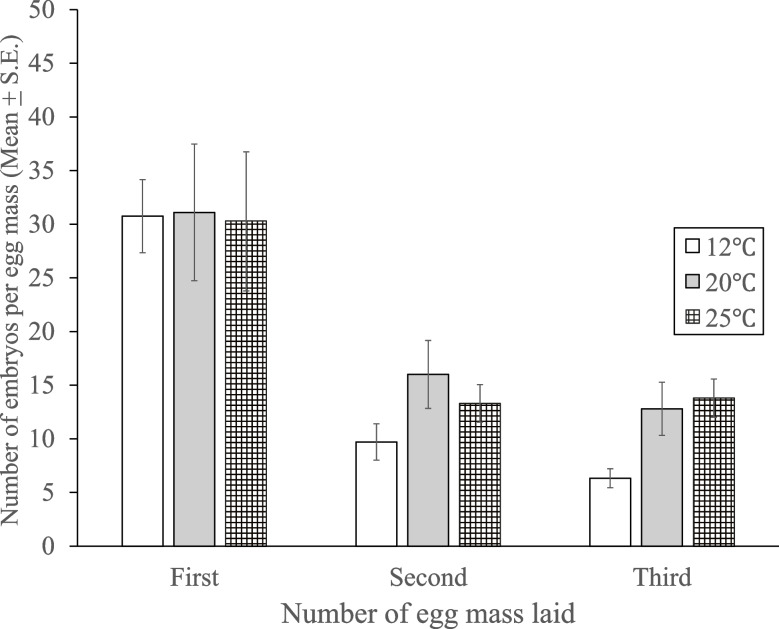
Mean (± S.E.) number of embryos produced per egg mass by *Physa gyrina* at three temperatures (12°C, 20 °C, and 25° C) in the first, second, and third egg mass laid by each snail. Number of snails producing egg masses from which embryos hatched are the same as those indicated in Fig. 1

There was no effect of temperature on the number of embryos per egg mass, and while there was an expected reduction in the number of egg masses laid over time because snails were not fed, the number of embryos per egg mass did not differ between temperature groups within each of the three egg-mass layings (Fig. 4).

#### Experiment #2: The effect of temperature and FLX on egg laying and hatching

**Fig. 5:**
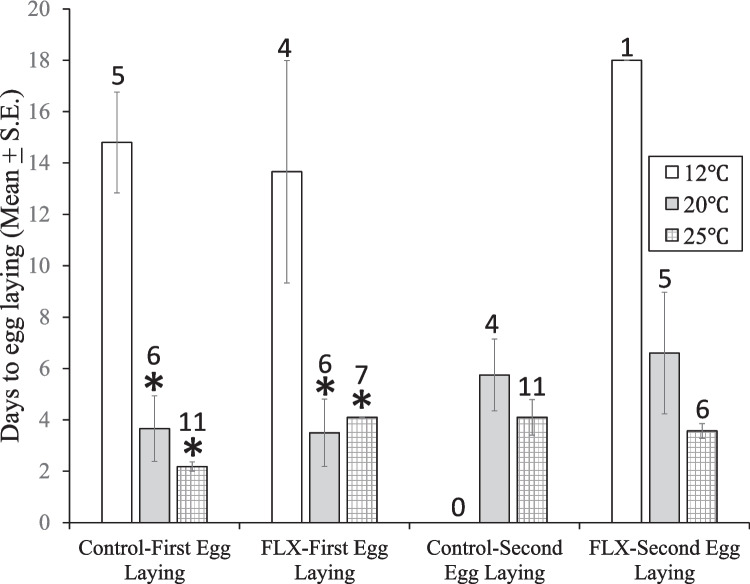
Mean (± S.E.) time in days for *Physa gyrina* snails to lay each of two egg masses at three temperatures (12°, 20°, and 25° C) and exposure to Fluoxetine (FLX, 3.45 µg/L). *: *p* < 0.01, Tukey’s post hoc test following one-way ANOVA of Box-Cox transformed data vs. 12°C. Number of snails laying eggs indicated above each bar

Exposure of *Physa gyrina* to the three experimental temperatures and FLX (3.45 µg/L) resulted in no significant combined effect on time to lay the first egg mass (2-way ANOVA, F (2, 33) = 1.57, *p* = 0.223, Table 3). There was a significant effect of FLX alone (F1, 33 = 11.52, *p* = 0.0018) and a significant effect of temperature alone (F 2,33) = 19.64, *p* < 0.0001). For the first egg laying, Tukey’s post hoc test showed significant pairwise comparisons between both 12 °C groups with each of the two 20 °C and 25 °C groups (Fig. 5).

**Table 3:** ANOVA table for mean time (days) to egg laying, and to egg hatching by embryos for the first egg mass in *Physa gyrina* when exposed to three temperatures (12°C, 20 °C, 25 °C) and antidepressant (with and without fluoxetine 3.45 µg/L). Data were Box-Cox transformed

**Days to egg laying**					
Source	SS	DF	MS	F	*p*-value
A (antidepressant)	0.22	1	0.22	11.52	**0.0018**
B (temperature)	0.75	2	0.38	19.64	**<0.0001**
A x B	0.06	2	0.03	1.57	0.223
Error	0.63	33	0.02		
Total	1.66	38			
**Days to egg hatching**					
Source	SS	DF	MS	F	*p*-value
A (antidepressant)	0.000001	1	0.000001	0.122	0.729
B (temperature)	0.0009	2	0.00048	55.60	**3.86e-11**
A x B	0.0000008	2	0.0000043	0.49	0.615
Error	0.00028	32	0.0000087		
Total﻿	0.00126	37	0.0000342

**Fig. 6:**
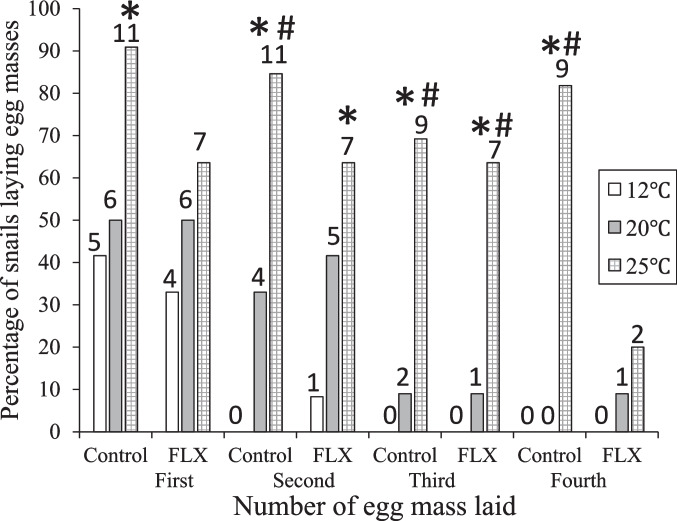
Percentage of snails laying each of four egg masses at three temperatures (12°C, 20 °C, and 25 °C) and exposure to Fluoxetine (FLX, 3.45 µg/L). *: *p* < 0.05 Fisher’s Exact test vs.12°C, #: *p* < 0.05 vs. 20°C. Number of snails laying eggs indicated above each bar. See Table 4 for data on egg masses 5–7

Snails in 25 °C consistently showed the highest percent frequency of egg laying across all groups (Fig. 6). There was no significant association between exposure to FLX and temperature for any of the first four egg masses laid (Cramer’s V, 0.04–0.52). When comparing the frequency of egg laying between snails exposed to FLX and the controls, there was no significant antidepressant effect. In addition, most snails in 12 °C, laid only one egg mass (1 out of 9 laid a second egg mass), whereas snails in 20 °C and 25 °C laid up to 7 egg masses (Table 4). When comparing the number of egg masses laid by snails in each temperature, those in the two warmest temperatures laid more multiple egg masses than snails in the 12 °C groups. Of the 42 possible comparisons of egg laying frequency between different temperatures, there were 16 significant differences observed, and 9 of the 16 comparisons were between 12 °C and 25 °C, the others were between 20 °C and 25 °C (Table 4).

**Table 4:** Results of Fisher’s Exact Tests for differences in the frequency of laying multiple egg masses comparing temperature groups in control water and fluoxetine (3.45 µg/L; FLX).  Numbers in parentheses (e.g. 4/12) are the number of snails laying egg masses out of the total number of snails in each group. Significant differences indicated in **bold**; NS: not significant

2 egg masses Control
12 °C (0/12) vs. 20 °C (4/12): NS
12 °C vs. 25 °C (11/11): ***p*** **= 0.0000003**
20 °C vs. 25 °C: ***p*** **= 0.001**
2 egg masses FLX
12 °C (1/12) vs. 20 °C (5/12): NS
12 °C vs. 25 °C (7/11): ***p*** **= 0.009**
20 °C vs. 25 °C: NS
3 egg masses Control
12 °C (0/12) vs. 20 °C (2/12): NS
12 °C vs. 25 °C (9/11): ***p*** **= 0.00006**
20 °C vs. 25 °C: ***p*** **= 0.003**
3 egg masses FLX
12 °C (0/12) vs 20 °C (1/12): NS
12 °C vs. 25 °C (7/11): ***p*** **= 0.001**
20 °C vs. 25 °C: ***p*** **= 0.009**
4 egg masses Control
12 °C (0/12) vs. 20 °C (1/11): NS
12 °C vs. 25 °C (9/11): ***p*** **= 0.00006**
20 °C vs. 25 °C: ***p*** **= 0.0006**
4 egg masses FLX
12 °C (0/12) vs. 20 °C (1/12): NS
12 °C vs. 25 °C (2/11): NS
20 °C vs. 25 °C: NS
5 egg masses Control
12 °C (0/12) vs 20 °C (0/12): NS
12 °C vs. 25 °C (7/12): ***p*** **= 0.004**
20 °C vs. 25 °C: ***p*** **= 0.004**
5 egg masses FLX
12 °C (0/12) vs. 20 °C (1/12): NS
12 °C vs. 25 °C (4/11): ***p*** **= 0.03**
20 °C vs. 25 °C: NS
6 egg masses Control
12 °C (0/12) vs. 20 °C (0/12): NS
12 °C (0/12) vs. 25 °C (5/11): ***p*** **= 0.01**
20 °C vs. 25 °C: ***p*** **= 0.01**
6 egg masses FLX
12 °C (0/12) vs. 20 °C (1/12): NS
12 °C vs. 25 °C (0/12): NS
20 °C vs. 25 °C: NS
7 egg masses Control
12 °C (0/12) vs. 20 °C (0/12): NS
12 °C vs. 25 °C (5/11): ***p*** **= 0.01**
20 °C vs. 25 °C: ***p*** **= 0.01**
7 egg masses FLX
12 °C (0/12) vs. 20 °C (1/12): NS
12 °C vs. 25 °C (0/12): NS
20 °C vs. 25 °C: NS

**Fig. 7:**
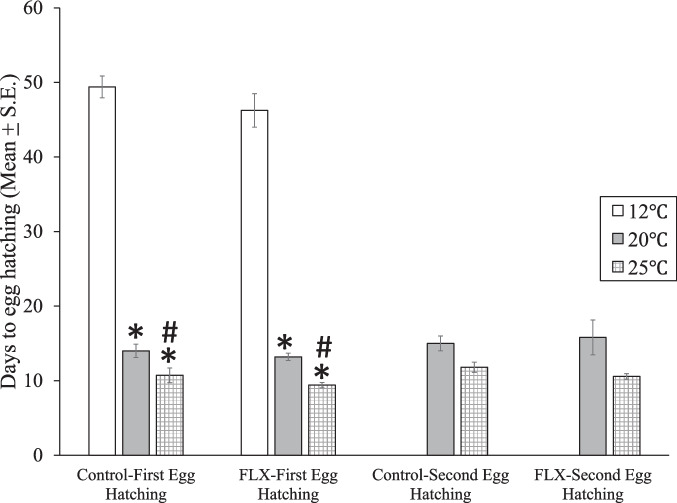
Mean (± S.E.) time in days for embryos of *Physa gyrina* to hatch from each of two egg masses at three temperatures (12°C, 20 °C, and 25 °C) and exposure to Fluoxetine (FLX, 3.45 µg/L). *: *p* < 0.01 vs. 12 °C, #: *p* < 0.01 vs.20°C, Tukey’s post hoc test following one-way ANOVA of Box-Cox transformed data. Number of egg masses are the same as the number of snails indicated in Fig. 5

The results for time to egg hatching were similar to those for egg laying. There was no significant combined effect of temperature and FLX (two-way Anova, F(2, 32) = 0.49, *p* = 0.615, Table 3) and there was a significant effect of temperature F(2, 32) = 55.6,

*p* = 3.86e^**−11**^ (Fig. 7, Table 3), but there was no significant effect of FLX alone F(1, 32) = 0.122, *p* = 0.729. Thus, warmer temperatures accelerated hatching time.

## Discussion

Climate change has had disruptive effects on the physiology of aquatic organisms. From increasing temperatures of the ocean and freshwater, and the concomitant decrease in pH and dissolved oxygen, these changes have resulted in a variety of physiological stressors at the population and individual levels, including survivorship and reproduction (Fernandez et al. 2022). The physiological adaptations that must evolve have therefore affected the distribution and abundance of organisms (Ehrlen and Morris 2015). In the present study, we examined the reproductive physiological effects of warmer temperatures and antidepressant exposure on the freshwater snail, *Physa gyrina*. Our major findings were that in each measure of reproductive end points, exposure to warmer temperatures induced faster and more frequent egg laying and faster hatching of embryos. When combined with an environmentally relevant concentration of FLX, an antidepressant shown to modulate reproduction in other *Physa* species*,* warmer temperatures were more important than antidepressant exposure at stimulating egg laying and embryo hatching, with FLX having only a minor effect on the timing of egg laying.

Previous studies on the closely related *P. acuta* showed varying and opposite effects of FLX on reproduction. Henry et al. (2022) found reduced egg laying in *P. acuta* at 32.7 ng/L FLX. By contrast, Sanchez-Arguello et al. (2009) reported that FLX at low concentrations (31.25 and 62.5 µg/L) stimulated reproduction, but reproduction was inhibited at 250 µg/L. In a different *Physa* species, Kimberly and Salice (2013) tested the effect of temperature and cadmium on hatching success and latency to hatching by embryos of *P. pomilia*. They found that high temperature (35°C in Texas, USA) without cadmium stimulated hatching, but when combined with cadmium, the 35 °C temperature reduced hatching success and increased the time to hatching. Reproduction in different species of freshwater snails has been tested for its sensitivity to higher temperatures. Leicht and Seppala (2019) found egg masses of *Lymnaea stagnalis* at high temperature (25°C) increased hatching success and shortened the time to hatching, but egg size and egg survival was reduced compared with controls at 15°C. Gust et al (2011) working with New Zealand mud snails found that temperature was more important than FLX exposure in affecting reproduction. While the antidepressant reduced embryo development, these authors found significantly more embryos produced at 16 °C than at 24 °C throughout the last half of their experiment (their Fig. 2A, B). In our experiments, we consistently showed greater release of embryos at 25 °C and 20 °C than at 12 °C, and little effect of FLX on egg laying time. In other recent studies testing the combined effects of temperature and antidepressant exposure, Auselbrook et al (2022) found that temperature limited the effect of antidepressants on life history traits of *Daphnia magna* and Fong et al. (2023) found a stronger effect of temperature than antidepressant exposure on the timing of metamorphosis in wood frog tadpoles (Fong et al. 2023).

Global surface temperature is predicted to increase from 1.8 to 4.0°C in the next 100 years (Priya et al. 2023). Warming climates will likely affect aquatic ectotherms such as snails by increasing their metabolism, which in turn would affect foraging time, growth, mate seeking time, as well as the timing of egg laying and egg hatching. As climate change is an environmental stressor and when combined with other stressors such as chemical pollutants, they will likely affect the distribution and abundance of these organisms. While negative impacts of climate change on aquatic species are thought to be more likely, some species could benefit from warming temperatures. The increase in snail egg laying as reported in our study could have positive consequences for the population size and future reproductive success. On the other hand, if the increase in egg laying occurs when food is insufficient, it could result in mortality of recently hatched juveniles.

The continued increase in global temperature raises concern for environmental stability and growth of populations of invasive species (Rahel and Olden 2008). Both *P. gyrina* and *P. acuta* are invasive species in Europe (Dobson 2004; Dillon 2024). Some of the ecological effects of invasive snails have been shown to be as vectors of disease parasites (Ebbs et al. 2018) and as competitors of native species (Nunez 2011; Orfao et al. 2024). The production of more eggs in a shorter time with faster hatching of embryos under warmer conditions, as we have found, could enable these invasive snails to establish themselves in new, invaded habitats more quickly. Our results highlight the increasing concern for the effects of climate change on the reproductive physiology of freshwater snails and for aquatic ectotherms, in general.

## Data Availability

The data are available on Figshare.com. 10.6084/m9.figshare.28100195.
